# Prognostic impact of pretreatment skeletal muscle index and CONUT score in diffuse large B-cell Lymphoma

**DOI:** 10.1186/s12885-023-11590-y

**Published:** 2023-11-06

**Authors:** Se-Il Go, Bong-Hoi Choi, Mi Jung Park, Sungwoo Park, Myoung Hee Kang, Hoon-Gu Kim, Jung Hun Kang, Eun Jeong Jeong, Gyeong-Won Lee

**Affiliations:** 1 Division of Hematology-Oncology, Department of Internal Medicine, Institute of Health Science, Gyeongsang National University Changwon Hospital, Gyeongsang National University College of Medicine, Changwon, Korea; 2 Department of Nuclear Medicine, Gyeongsang National University Hospital, Gyeongsang National University College of Medicine, Jinju, Korea; 3 Department of Radiology, Institute of Health Science, Gyeongsang National University Hospital, Gyeongsang National University College of Medicine, Jinju, Korea; 4 Division of Hematology-Oncology, Department of Internal Medicine, Institute of Health Science, Gyeongsang National University Hospital, Gyeongsang National University College of Medicine, Gangnam-ro 79 Jinju, Jinju, 52727 Korea

**Keywords:** Lymphoma, Diffuse large B-cell, Cachexia, Malnutrition, Sarcopenia, Controlling nutritional status

## Abstract

**Background:**

Although the prognostic value of the Controlling Nutritional Status (CONUT) score in diffuse large B-cell lymphoma (DLBCL) has been reported in several previous studies, its clinical relevance for the presence of sarcopenia has not been assessed.

**Methods:**

In this study, 305 DLBCL patients were reviewed. They were categorized into normal/mild (*n* = 219) and moderate/severe (*n* = 86) CONUT groups. Sarcopenia was assessed using the L3-skeletal muscle index measured by baseline computed tomography imaging. Based on CONUT score and sarcopenia, patients were grouped: A (normal/mild CONUT and no sarcopenia), B (either moderate/severe CONUT or sarcopenia, but not both), and C (both moderate/severe CONUT and sarcopenia).

**Results:**

The moderate/severe CONUT group showed higher rates of ≥ grade 3 febrile neutropenia, thrombocytopenia, non-hematologic toxicities, and early treatment discontinuation not related to disease progression, compared to the normal/mild CONUT group. The moderate/severe CONUT group had a lower complete response rate (58.1% vs. 80.8%) and shorter median overall survival (18.5 vs. 162.6 months) than the normal/mild group. Group C had the poorest prognosis with a median survival of 8.6 months, while groups A and B showed better outcomes (not reached and 60.1 months, respectively). Combining CONUT score and sarcopenia improved the predictive accuracy of the Cox regression model (C-index: 0.763), compared to the performance of using either CONUT score (C-index: 0.754) or sarcopenia alone (C-index: 0.755).

**Conclusions:**

In conclusion, the moderate/severe CONUT group exhibited treatment intolerance, lower response, and poor prognosis. Additionally, combining CONUT score and sarcopenia enhanced predictive accuracy for survival outcomes compared to individual variables.

**Supplementary Information:**

The online version contains supplementary material available at 10.1186/s12885-023-11590-y.

## Introduction

Diffuse large B-cell lymphoma (DLBCL) is the most common type of non-Hodgkin lymphoma in adults [1]. In the past two decades, rituximab plus cyclophosphamide, doxorubicin, vincristine, and prednisolone (R-CHOP) has been a standard frontline treatment in DLBCL patients [2]. As a prognostic marker of DLBCL, the International Prognostic Index (IPI) and its variant have been widely used [3, 4].

Cancer cachexia was suggested as an emerging prognostic factor in DLBCL [5]. Cancer cachexia is defined by two main components, sarcopenia and malnutrition [6]. Sarcopenia is characterized as a progressive skeletal muscle disorder with the accelerated loss of muscle mass and function [7]. Because sarcopenia is known to be associated with intolerance to R-CHOP therapy, dose adjustment and intensive supportive care should be considered for patients with sarcopenia [8]. Additionally, it has been reported that sarcopenia is associated with poor survival outcomes in DLBCL [8–10].

Several indices reflecting patients’ nutritional status could help to predict their response to chemotherapy [11, 12]. Among them, the Controlling Nutritional Status (CONUT) score, which considers serum albumin level, total lymphocyte count, and total cholesterol level, has been known to be associated with the prognosis of DLBCL patients [12, 13]. However, it is unclear whether the prognostic role of the CONUT score is independently significant or affected by the presence of sarcopenia in DLBCL because there is a strong relationship between sarcopenia and nutritional status in cancer and non-cancer patients [14, 15]. Furthermore, systemic inflammation, a fundamental process of cancer cachexia, is closely related to increased CONUT scores and sarcopenia [16–21]. Understanding the relationship of the CONUT score with sarcopenia may provide additional insights into the prognostic value of this score. Therefore, we conducted this study to determine the prognostic value of the CONUT score according to the third lumbar skeletal muscle index (L3-SMI) and to assess whether a combined model of the CONUT score and the L3-SMI has improved accuracy to predict prognosis compared with the CONUT score or the L3-SMI alone in DLBCL patients who received R-CHOP immunochemotherapy.

## Methods

### Patients and CONUT score

The study retrospectively reviewed the medical records of all consecutive DLBCL patients who received R-CHOP immunochemotherapy as a frontline treatment between January 26, 2004, and June 28, 2022, at Gyeongsang National University Hospital. To calculate the CONUT score, we used the latest laboratory data gathered at most seven days before the start of treatment. The CONUT score was calculated as the sum of the following criteria: (1) serum albumin (g/dL) ≥ 3.5, 3.0–3.49, 2.5–2.99, and < 2.5 as 0, 2, 4, and 6 points; (2) total lymphocyte counts (/mL) > 1,600, 1,200–1,599, 800–1,199, and < 800 as 0, 1, 2, and 3 points; (3) total cholesterol (mg/dL) > 180, 140–180, 100–139, and < 100 as 0, 1, 2, and 3 points, respectively. In addition, patients were classified into normal (CONUT 0–1), mild (CONUT 2–4), moderate (CONUT 5–8), and severe (CONUT 9–12) groups [22]. Patients in whom each parameter of CONUT and baseline computed tomography (CT) imaging were not measured and who were diagnosed with double primary malignancy were excluded from the analysis.

### Definitions of clinical variables

CT imaging was used to measure the L3-SMI. The sex-specific cutoffs for L3-SMI (52.4 cm^2^/m^2^ for men and 38.5 cm^2^/m^2^ for women) were used to stratify the patients into low and high L3-SMI groups [23]. The Lugano classification lymphoma response criteria were used to evaluate the tumor response [24]. Treatment-related toxicity was assessed using NCI Common Terminology Criteria for Adverse Events version 5.0. The cell-of-origin was determined using the Hans criteria [25]. Dose reduction of R-CHOP treatment at the first cycle was, at the discretion of the treating physician, typically considered for elderly patients with poor performance status and comorbidities. Further dose reduction was considered during treatment period in response to severe adverse events, delayed recovery from adverse events, or patient preference. The relative dose intensity (RDI) was calculated as the percentage of the total dose administered for each drug concerning the planned dose. Early treatment discontinuation refers to any premature termination of treatment unrelated to disease progression. The definition of treatment-related mortality included any death related to R-CHOP treatment, regardless of the time of occurrence, and any death within a month of R-CHOP treatment, although not because of disease progression.

### Statistical analysis

Group comparisons of categorical variables were performed using the chi-square test or Fisher’s exact test. In the case of continuous variables, the Mann-Whitney U test was used. Overall survival (OS) was calculated as the duration from the start of treatment to death or last follow-up. Progression-free survival (PFS) was measured from the start of treatment to death, progression during or after treatment, or until the last follow-up. The Kaplan-Meier method was used to plot survival curves of OS and PFS, and the log-rank test was used to compare the survival distribution between curves. Multivariate analysis for OS and PFS was performed using Cox proportional regression models. Because there was no death and disease progression in patients with low NCCN-IPI, the low and low-intermediate category of NCCN-IPI was combined when the multivariate analysis was performed. The predictability of the prognostic model was assessed by calculating Harrell’s C-index. We conducted 10-fold cross-validation and 1,000-bootstrap internal validation to validate the Cox regression models. Variables with *p*-values less than 0.1 in univariate analysis were entered into the multivariate model, and factors with *p*-values less than 0.05 were considered significant. All analyses were conducted using the Stata software version 16.1 (Stata Corp, College Station, TX, USA) and R software version 4.3.1 (R Foundation for Statistical Computing, Vienna, Austria).

## Results

### Patient characteristics

The total number of patients included in the study was 305. Given the distribution of survival curves (Supplementary Fig. 1), patients were classified into normal/mild (*n* = 219) and moderate/severe (*n* = 86) CONUT groups. There were differences in patient characteristics between the normal/mild and moderate/severe CONUT groups (Table 1). The moderate/severe CONUT group was associated with old age, poor Eastern Cooperative Oncology Group (ECOG) performance status, B-symptoms, advanced Ann Arbor stage, higher NCCN-IPI, bone marrow involvement, extranodal disease, and higher lactate dehydrogenase and C-reactive protein levels compared with the normal/mild CONUT group. Median C-reactive protein levels were 5.1 mg/L (IQR, 1.6–26.5) and 10.1 mg/L (IQR, 2.7–51.35) in the high and low L3-SMI groups, respectively (*p* = 0.011). There were no statistically significant differences in sex, bulky disease, cell-of-origin, and L3-SMI.

**Table 1:** Patients characteristics

Characteristics	Normal/mild CONUT (*n* = 219)	Moderate/severe CONUT (*n* = 86)	*P*
Sex			0.866
Men	125 (57.1)	50 (58.1)	
Women	94 (42.9)	36 (41.9)	
Age, median (IQR), years	63 (50–72)	70 (63–75)	< 0.001
ECOG PS			< 0.001
0–1	179 (81.7)	44 (51.2)	
2–3	40 (18.3)	42 (48.8)	
Symptom stage			0.001
A	191 (87.2)	61 (70.9)	
B	28 (12.8)	25 (29.1)	
Ann Arbor Stage			< 0.001
I–II	110 (50.2)	17 (19.8)	
III–IV	109 (49.8)	69 (80.2)	
NCCN-IPI			< 0.001
Low	26 (11.9)	1 (1.2)	
Low-intermediate	93 (42.5)	12 (14.0)	
High-intermediate	75 (34.3)	35 (40.7)	
High	25 (11.4)	38 (44.2)	
Bone marrow involvement			0.031
Presence	23 (10.5)	17 (19.8)	
Absence	196 (89.5)	72 (80.2)	
Extranodal disease			0.008
Presence	122 (55.7)	62 (72.1)	
Absence	97 (44.3)	24 (27.9)	
Bulky disease			0.594
Presence	40 (18.3)	18 (20.9)	
Absence	179 (81.7)	68 (79.1)	
Lactate dehydrogenase normalized			< 0.001
≤1	105 (48.0)	14 (16.3)	
>1 to ≤3	100 (45.7)	51 (59.3)	
> 3	14 (6.4)	21 (24.4)	
Cell-of-origin			0.142
GCB	48 (21.9)	12 (14.0)	
Non-GCB	102 (46.6)	50 (58.1)	
Not available	69 (31.5)	24 (27.9)	
C-reactive protein, median (IQR), mg/L^a^	3.5 (1.3–12.8)	39.5 (13.4–72.8)	< 0.001
L3-SMI			0.248
Low	91 (41.6)	42 (48.8)	
High	128 (58.5)	44 (51.2)	

^a^The value could be obtained in 232 out of 305 patients

*CONUT* Controlling Nutritional Status, *IQR* interquartile range, *NCCN-IPI* National Comprehensive Cancer Network International Prognostic Index, *GCB* germinal center B cell-like, *SMI* skeletal muscle index

Variables are presented as number (%) or median (IQR)

### Treatment-related toxicity and treatment response

Treatment-related toxicity could be evaluated in a total of 305 patients (Table 2). Dose reduction of any drug from the first cycle (30.2% vs. 16.4%) was more frequent in the moderate/severe CONUT group. RDIs of cyclophosphamide and doxorubicin were also lower in the moderate/severe CONUT group than in the normal/mild CONUT group. Nevertheless, the incidences of ≥grade 3 thrombocytopenia (45.4% vs. 24.2%), febrile neutropenia (44.2% vs. 23.7%), and non-hematological toxicity (41.9% vs. 29.2%) were significantly higher in the moderate/severe CONUT group than in the normal/mild CONUT group. Furthermore, the rate of early treatment discontinuation (30.2% vs. 13.2%) unrelated to disease progression was also higher in the moderate/severe CONUT group than in the normal/mild CONUT group.

**Table 2:** Treatment-related toxicity

	Normal/mild CONUT (*n* = 219)	Moderate/severe CONUT (*n* = 86)	*P*
Hematologic toxicity, grade ≥3			
anemia	39 (17.8)	23 (26.7)	0.081
thrombocytopenia	53 (24.2)	39 (45.4)	< 0.001
neutropenia	160 (73.1)	71 (82.6)	0.082
febrile neutropenia	52 (23.7)	38 (44.2)	< 0.001
Non-hematologic toxicity, grade ≥3^a^	64 (29.2)	36 (41.9)	0.034
asthenia	27 (12.3)	12 (14.0)	0.702
Infection^b^	17 (7.8)	6 (7.0)	0.815
diarrhea and enterocolitis	7 (3.2)	5 (5.8)	0.329
peripheral sensory neuropathy	5 (2.3)	6 (7.0)	0.080
thromboembolic event	7 (3.2)	5 (5.8)	0.329
Treatment-related mortality	15 (6.9)	9 (10.5)	0.291
Dose reduction at the first cycle	36 (16.4)	26 (30.2)	0.007
Dose reduction after the first cycle	82 (37.4)	31 (36.1)	0.820
RDI of cyclophosphamide, %			0.0495
mean (SD)	91.1 (13.4)	87.0 (15.9)	
median (IQR)	100 (85–100)	94.5 (75–100)	
RDI of doxorubicin, %			0.063
mean (SD)	90.6 (14.2)	86.8 (15.9)	
median (IQR)	100 (83–100)	94 (75–100)	
Early treatment discontinuation	29 (13.2)	26 (30.2)	0.001

^a^Toxicities with an incidence of > 3% in all patients are specified

^b^Included are lung/soft tissue/urinary tract infections, shingles, and sepsis

*CONUT* Controlling Nutritional Status, *RDI* Relative dose intensity, *SD* Standard deviation, *IQR* Interquartile range

Variables are presented as number (%), mean (SD), or median (IQR)

Treatment response could be evaluated for 287 out of 305 patients (Table 3). The complete response (CR) rates were 58.1% and 80.8% (*p* < 0.001) in moderate/severe and normal/mild CONUT groups, respectively. CR rate tends to be lower according to the degree of CONUT score. When the 55 patients who discontinued treatment early for reasons unrelated to disease progression were excluded from the analysis, CR rates were 76.7% and 87.4% in the moderate/severe and normal/mild CONUT groups, respectively (*p* = 0.044).

**Table 3:** Treatment response according to the CONUT score

	Normal	Mild	Moderate	Severe
Complete response	70 (87.5)	107 (77.0)	41 (60.3)	9 (50.0)
Partial response	7 (8.8)	22 (15.8)	17 (25.0)	6 (33.3)
Stable disease	0	4 (2.9)	1 (1.5)	0
Progressive disease	0	2 (1.4)	1 (1.5)	0
Not evaluable	3 (3.8)	4 (2.9)	8 (11.8)	3 (16.7)

*CONUT* Controlling Nutritional Status

Variables are presented as number (%)

### Survival

**Fig. 1:**
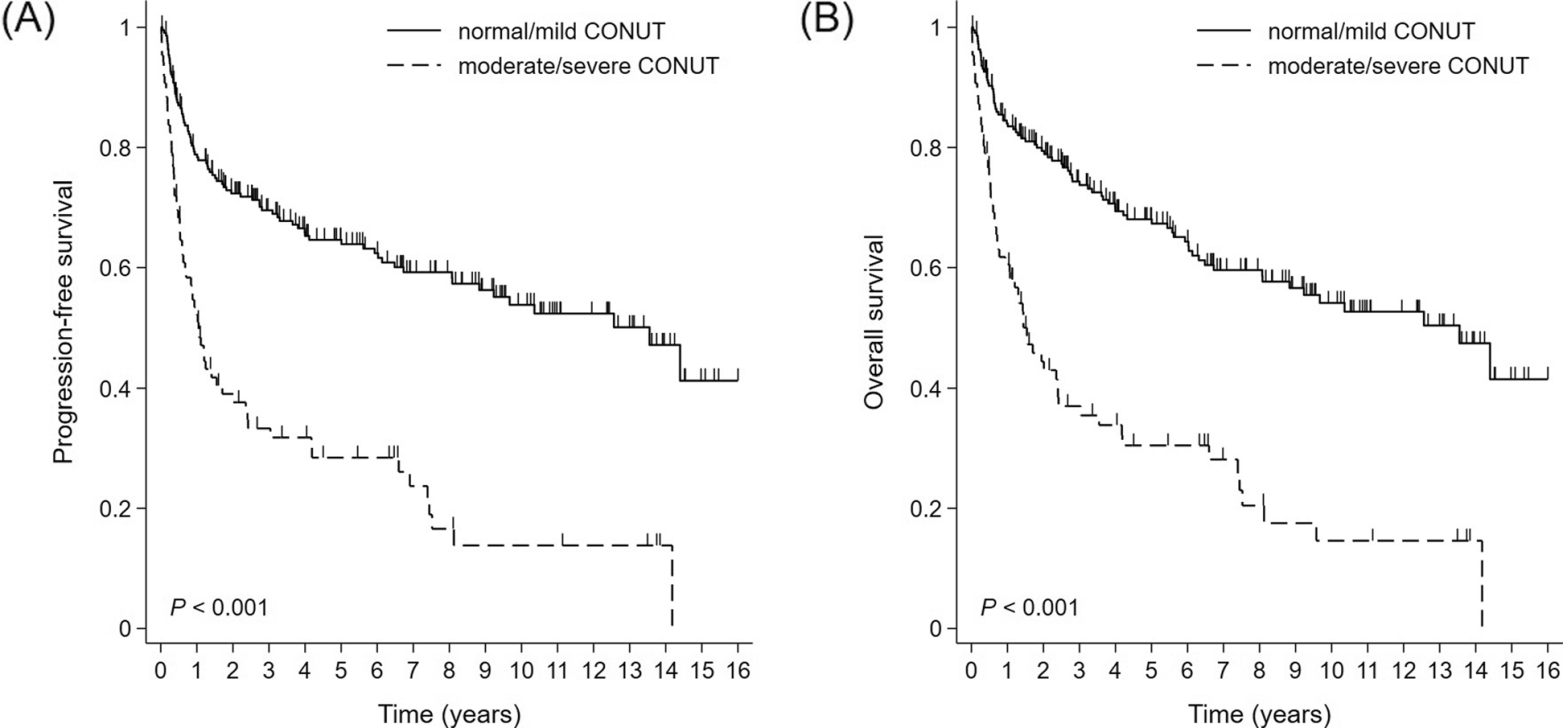
Kaplan-Meier survival curves for (**A**) progression-free survival and (**B**) overall survival

**Fig. 2:**
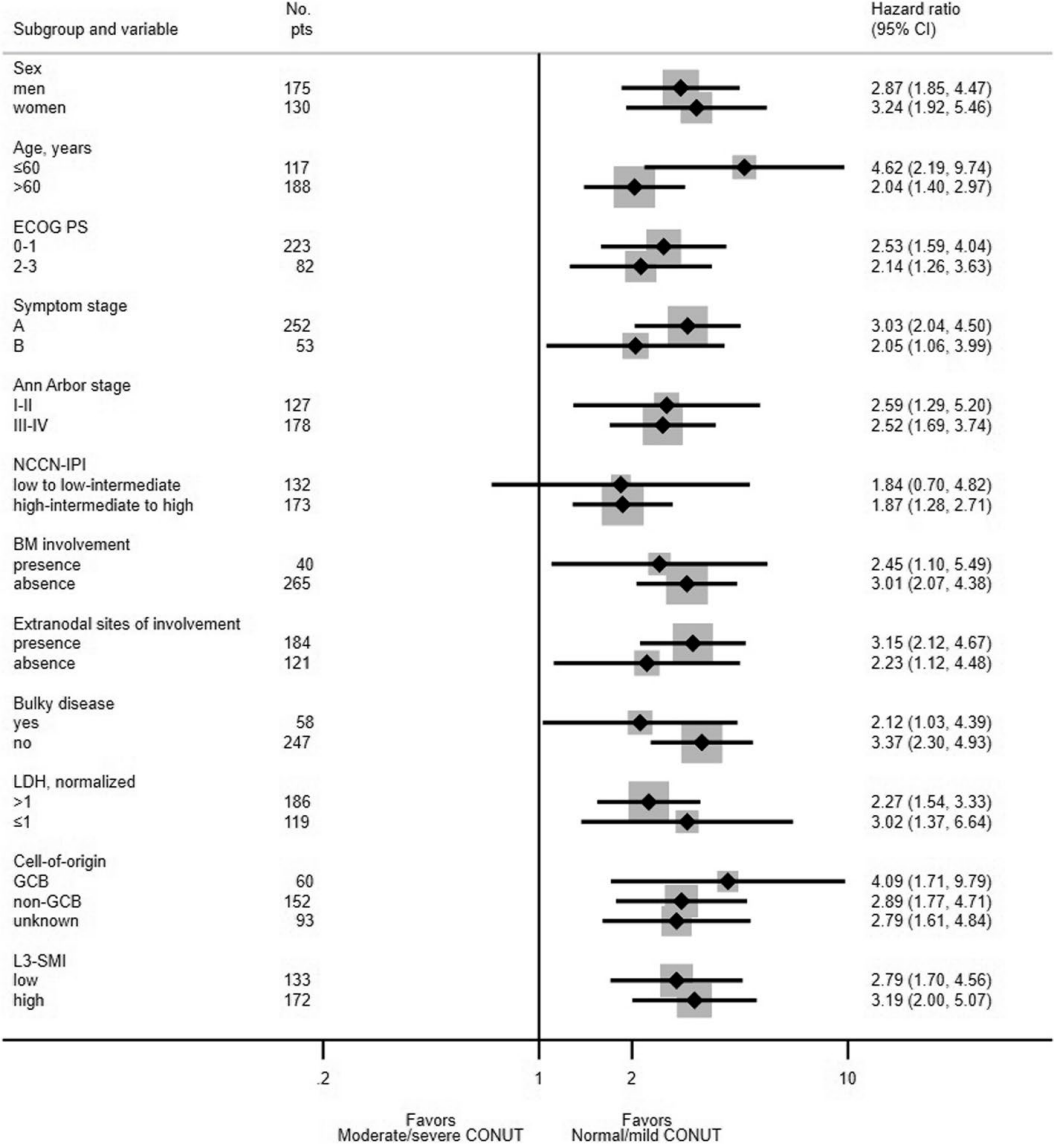
Forest plot for overall survival by subgroup

During the analysis, mortality occurred in 61 out of 86 moderate/severe CONUT groups and 82 out of 219 normal/mild CONUT groups, respectively. At the end of the median follow-up duration of 106 months, the median PFS was 12.6 months (95% CI, 7.5 to 20.5 months) in the moderate/severe CONUT group and 162.6 months (95% CI, 97.0 months to not determined) in the normal/mild CONUT group (*p* < 0.001; Fig. 1A). The median OS duration was 18.5 months (95% CI, 11.3 to 28.8 months) in the moderate/severe CONUT group and 162.6 months (95% CI, 97.0 months to not determined) in the normal/mild CONUT group (*p* < 0.001; Fig. 1B). In nearly all subgroup categories, subgroup analysis showed worse survival outcomes in the moderate/severe CONUT group (Fig. 2).

**Fig. 3:**
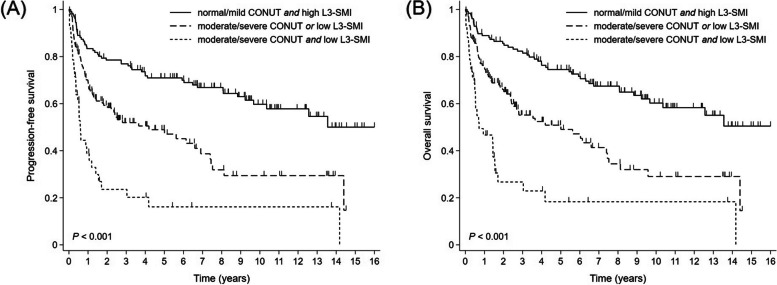
Kaplan-Meier survival curves for (**A**) progression-free survival and (**B**) overall survival according to the CONUT score and the level of the L3-SMI

Next, we assessed the prognostic impact of the combined model incorporating the CONUT score and the level of the L3-SMI (CONUT-SMI category). Patients were reclassified as follows: group A, both normal/mild CONUT score and high L3-SMI (*n* = 128); group B, either moderate/severe CONUT score or low L3-SMI (*n* = 135), but not both; and group C, both moderate/severe CONUT score and low L3-SMI (*n* = 42). The median PFS was not reached in group A (95% CI, 116.1 months to not determined), 50.3 months in group B (95% CI, 24.1 months to 82.9 months), and 7.3 months in group C (95% CI 4.4 months to 14.3 months) (*p* < 0.001; Fig. 3A). The median OS was not reached in group A (95% CI, 116.1 months to not determined), 60.1 months in group B (95% CI, 30.4 months to 89.3 months), and 8.6 months in group C (95% CI 5.9 months to 18.5 months; *p* < 0.001; Fig. 3B). While patients in group A and B who received treatment without dose reduction at the beginning of treatment had superior prognoses, administering the full dose of R-CHOP treatment did not lead to improved outcomes in group C (Supplementary Fig. 2).

The results from the multivariate Cox regression analyses, as presented in Table 4, reveal that a moderate/severe CONUT score independently serves as a prognostic factor for both PFS (Hazard Ratio [HR] 1.499, 95% confidence interval [CI] 1.040 to 2.159, *p* = 0.030) and OS (HR 1.470, 95% CI 1.011 to 2.136, *p* = 0.044). When the CONUT-SMI category is used instead of evaluating each index separately, while NCCN-IPI remains a top-performing predictor, the CONUT-SMI category also demonstrates strong predictive performance, with a progressively worsening prognosis observed from group A (best outcome) to group C (worst outcome). The bootstrap internal validation confirms the statistical significance of both models, as detailed in Supplementary Tables 1A and B. These two Cox regression models, adjusted for B-symptoms and NCCN-IPI, consistently demonstrate superior predictive accuracy with C-indices of 0.763 and 0.762, respectively, compared to models using the CONUT score alone (C-index = 0.754) or L3-SMI alone (C-index = 0.755). In the 10-fold cross-validation, both models, which include both the CONUT score and L3-SMI as separate variables and the Cox regression model that incorporates the CONUT-SMI category (comprising groups A, B, and C), maintained strong predictive accuracy with an optimism-corrected C-index of 0.760, indicating results in close proximity to their original C-index values of 0.763 and 0.762, respectively.

**Table 4:** Univariate and multivariate Cox regression for progression-free and overall survival

Progression-free survival	Univariate		Multivariate (1)^a^		Multivariate (2)^b^	
	HR (95% CI)	*P*	HR (95% CI)	*P*	HR (95% CI)	*P*
Sex (men vs. women)	1.165 (0.842–1.612)	0.357				
Symptom stage (B vs. A)	2.357 (1.630–3.407)	< 0.001	1.555 (1.059–2.282)	0.024	1.556 (1.066–2.273)	0.022
NCCN-IPI						
Low to low-intermediate	Ref.		Ref.		Ref.	
High-intermediate	5.476 (3.468–8.645)	< 0.001	4.754 (2.965–7.622)	< 0.001	4.707 (2.939–7.537)	< 0.001
High	11.289 (6.858–18.584)	< 0.001	8.190 (4.694–14.290)	< 0.001	8.168 (4.732–14.101)	< 0.001
Bone marrow involvement (yes vs. no)	2.387 (1.591–3.579)	< 0.001	1.109 (0.721–1.704)	0.637	1.111 (0.724–1.706)	0.630
Bulky disease (yes vs. no)	1.108 (0.745–1.646)	0.613				
Cell-of-origin						
GCB	Ref.					
Non-GCB	1.086 (0.679–1.738)	0.730				
Unknown	0.975 (0.592–1.605)	0.921				
L3-SMI (low vs. high)	1.834 (1.322–2.544)	< 0.001	1.561 (1.113–2.189)	0.010		
CONUT						
Normal to mild	Ref.		Ref.			
Moderate to severe	2.973 (2.142–4.126)	< 0.001	1.499 (1.040–2.159)	0.030		
CONUT-SMI category						
Group A	Ref.				Ref.	
Group B	2.231 (1.535–3.243)	< 0.001			1.632 (1.106–2.407)	0.014
Group C	5.091 (3.212–8.068)	< 0.001			2.314 (1.392–3.844)	0.001
Overall survival	Univariate		Multivariate			
	HR (95% CI)	*P*	HR (95% CI)	*P*		
Sex (men vs. women)	1.166 (0.835–1.629)	0.368				
Symptom stage (B vs. A)	2.279 (1.561–3.325)	< 0.001	1.556 (1.051–2.302)	0.027	1.544 (1.048–2.276)	0.028
NCCN-IPI						
Low to low-intermediate	Ref.		Ref.		Ref.	
High-intermediate	4.932 (3.097–7.854)	< 0.001	4.369 (2.703–7.062)	< 0.001	4.266 (2.644–6.883)	< 0.001
High	12.775 (7.702–21.188)	< 0.001	9.687 (5.510–17.030)	< 0.001	9.486 (5.460–16.480)	< 0.001
Bone marrow involvement (yes vs. no)	2.264 (1.480–3.462)	< 0.001	1.030 (0.655–1.619)	0.899	1.037 (0.660–1.630)	0.875
Bulky disease (yes vs. no)	1.142 (0.763–1.711)	0.518				
Cell-of-Origin						
GCB	Ref.					
Non-GCB	1.067 (0.653–1.744)	0.795				
Unknown	0.982 (0.585–1.649)	0.946				
L3-SMI (low vs. high)	1.925 (1.373–2.699)	< 0.001	1.670 (1.178–2.369)	0.004		
CONUT						
Normal to mild	Ref.		Ref.			
Moderate to severe	3.023 (2.159–4.232)	< 0.001	1.470 (1.011–2.136)	0.044		
CONUT-SMI category						
Group A	Ref.				Ref.	
Group B	2.333 (1.590–3.423)	< 0.001			1.721 (1.157–2.561)	0.007
Group C	5.430 (3.377–8.733)	< 0.001			2.423 (1.436–4.087)	0.001

^a^The analysis excludes the CONUT + L3-SMI combined model

^b^Neither the CONUT nor L3-SMI individual models are included in the analysis

*HR* hazard ratio, *95% CI* 95% confidence interval, *NCCN-IPI* National Comprehensive Cancer Network International Prognostic Index, *GCB* germinal center B cell-like, *SMI* skeletal muscle index, *CONUT* Controlling Nutritional Status

## Discussion

This study showed that the moderate/severe CONUT score was associated with worse survival outcomes in DLBCL patients treated with frontline R-CHOP treatment, regardless of the level of the L3-SMI. Although there is a noticeable association between the CONUT score and recognized prognostic determinants in DLBCL, our subgroup and multivariate analyses underscore the CONUT score’s independent predictive potency. Despite its close association with factors like age, ECOG performance status, and others, the CONUT score’s distinct value remains evident. The moderate/severe CONUT group more frequently experienced ≥ grade 3 hematologic and non-hematologic treatment-related toxicities and early treatment discontinuation than the normal/mild CONUT group. The intolerance to R-CHOP treatment within the moderate/severe CONUT group might be associated with a lower treatment response rate and poorer survival outcomes. While it is considered that more frequent dose reductions and a lower RDI of the drug could potentially impact treatment response, it is worth noting that intolerance to treatment may exacerbate in the moderate/severe group if adjustments to the R-CHOP dosage are not made. In fact, there is no impact of the R-CHOP treatment dose on survival in group C in this study, whereas dose reduction was associated with inferior survival outcomes in groups A and B. Additionally, the lower CR rate persisted in the moderate/severe CONUT group even among patients who did not experience early treatment discontinuation for reasons unrelated to disease progression. This observation suggests that factors beyond early treatment discontinuation and intolerance to treatment, such as an inherent resistance to treatment, may partly contribute to the lower CR rate in the moderate/severe CONUT group.

Many different approaches have demonstrated the utility of the CONUT score in predicting survival outcomes in various malignancies, including DLBCL [26–30]. However, no study assessed the relationship between CONUT score and sarcopenia, namely the clinical impact of CONUT score according to sarcopenia status in DLBCL. In advanced urothelial carcinoma, incorporating the CONUT score or sarcopenia into well-known prognostic models increased the prognostic value of each model. The model performance to predict survival was highest when both the CONUT score and sarcopenia were incorporated into the model [31]. Several studies reported a close relationship between CONUT score and sarcopenia in various conditions [32–34]. By contrast, other studies reported the low predictability of CONUT score on sarcopenia [35, 36]. In our study, there was no difference in the proportion of sarcopenic patients between the normal/mild and moderate/severe CONUT groups. Both the L3-SMI and CONUT score were independent prognostic factors for survival. The prognostic value of the CONUT score was consistent regardless of the level of the L3-SMI. In addition, the predictive accuracy of the CONUT score was increased when the combined model incorporating both the CONUT score and the L3-SMI was used compared with the model including either the CONUT score or the L3-SMI alone. While malnutrition and sarcopenia have common features in terms of etiology and pathogenesis, including systemic inflammation and anorexia [37, 38], there are differences in their clinical manifestation and the dominant contributing factors for each condition. An imbalance between energy intake and expenditure primarily contributes to malnutrition, whereas it plays a lesser role in sarcopenia [37, 38]. Reduced activity and increased loss of neural fiber are dominant factors contributing to sarcopenia, but are less likely to contribute to malnutrition [38]. It was reported that the coexistence of malnutrition and sarcopenia resulted in a negative clinical impact in elderly patients with gastric cancer [39]. These findings indicate that assessing both malnutrition and sarcopenia may help to predict the prognosis of cachexic patients more accurately. Patients with both moderate/severe CONUT score and sarcopenia had a grave prognosis, and their prognosis was not improved by dose adjustment of R-CHOP treatment. Therefore, an alternative treatment strategy with intensive supportive care may be needed in this population.

This study has several limitations. First, the study’s sample size of only 305 DLBCL patients may limit the generalization of the results. In addition, the study was conducted at a single institution and may not accurately represent other geographic locations or ethnicities. Second, the study’s retrospective nature, where data was obtained from medical records, is also a drawback. While we attempted to mitigate selection bias through internal validation via bootstrap and cross-validation methods, there is a need for prospective studies or studies with external validation cohorts to further support the findings. Third, using baseline CT imaging alone to measure the L3-SMI may not accurately assess changes in muscle mass over time and did not assess functional aspects of sarcopenia, such as muscle power or physical performance. Fourth, there is the lack of information regarding the use of corticosteroids before the first cycle of R-CHOP treatment. Because corticosteroids can influence the lymphocyte count, which is one of the factors used to calculate the CONUT score, their use may affect the accuracy of the study’s findings. Finally, we did not investigate the underlying biological mechanisms that connect the CONUT score and the L3-SMI with clinical outcomes in patients with DLBCL.

In conclusion, the findings of this study suggest that the CONUT score is a valuable and independent prognostic indicator for DLBCL patients treated with R-CHOP. Its predictive value is stronger when the combined model incorporating both the CONUT score and the L3-SMI is used. A prospective study design with a larger cohort of DLBCL patients is warranted to provide more comprehensive data on malnutrition, sarcopenia, and clinical outcomes, leading to better management for those with moderate/severe CONUT score and sarcopenia having an extremely poor prognosis.

### Supplementary Information

**Additional file 1: Supplementary Fig. 1. **Kaplan-Meier survival curves for overall survival according to the CONUT score. **Supplemental Fig. 2.** Kaplan-Meier survival curves for overall survival according to the dose reduction from the first cycle and CONUT-SMI category in (A) group A, (B) group B, and (C) group C, respectively. *CONUT* Controlling Nutritional Status, *SMI* skeletal muscle index. **Supplementary Table 1A. **Bootstrap internal validation (1,000 replications) results for multivariate Cox regression analysis of progression-free and overall survival. *HR* hazard ratio, *SE* standard error, *95% CI* 95% confidence interval, *NCCN-IPI* National Comprehensive Cancer Network International Prognostic Index, *SMI* skeletal muscle index, *CONUT* Controlling Nutritional Status. **Supplementary Table 1B. **Bootstrap internal validation (1,000 replications) results for multivariate Cox regression analysis of progression-free and overall survival. *HR* hazard ratio, *SE* standard error, *95% CI* 95% confidence interval, *NCCN-IPI* National Comprehensive Cancer Network International Prognostic Index, *CONUT* Controlling Nutritional Status, *SMI* skeletal muscle index.

## Data Availability

The datasets used and/or analyzed during the current study are available from the corresponding author on reasonable request.
