# Supplementary material for: Prognostic impact of pretreatment skeletal muscle index and CONUT score in diffuse large B-cell Lymphoma
Source: BMC Cancer. 2023 Nov 6;23:1071. doi: 10.1186/s12885-023-11590-y (PMC10629181; doi:10.1186/s12885-023-11590-y)
Supplement: Supplementary file 1 — Additional file 1: Supplementary Fig. 1. Kaplan-Meier survival curves for overall survival according to the CONUT score. Supplemental Fig. 2. Kaplan-Meier survival curves for overall survival according to the dose reduction from the first cycle and CONUT-SMI category in (A) group A, (B) group B, and (C) group C, respectively. CONUT Controlling Nutritional Status, SMI skeletal muscle index. Supplementary Table 1A. Bootstrap internal validation (1,000 replications) results for multivariate Cox regression analysis of progression-free and overall survival. HR hazard ratio, SE standard error, 95% CI 95% confidence interval, NCCN-IPI National Comprehensive Cancer Network International Prognostic Index, SMI skeletal muscle index, CONUT Controlling Nutritional Status. Supplementary Table 1B. Bootstrap internal validation (1,000 replications) results for multivariate Cox regression analysis of progression-free and overall survival. HR hazard ratio, SE standard error, 95% CI 95% confidence interval, NCCN-IPI National Comprehensive Cancer Network International Prognostic Index, CONUT Controlling Nutritional Status, SMI skeletal muscle index. [file 12885_2023_11590_MOESM1_ESM.docx]

**Supplementary Fig. 1** Kaplan-Meier survival curves for overall survival according to the CONUT score


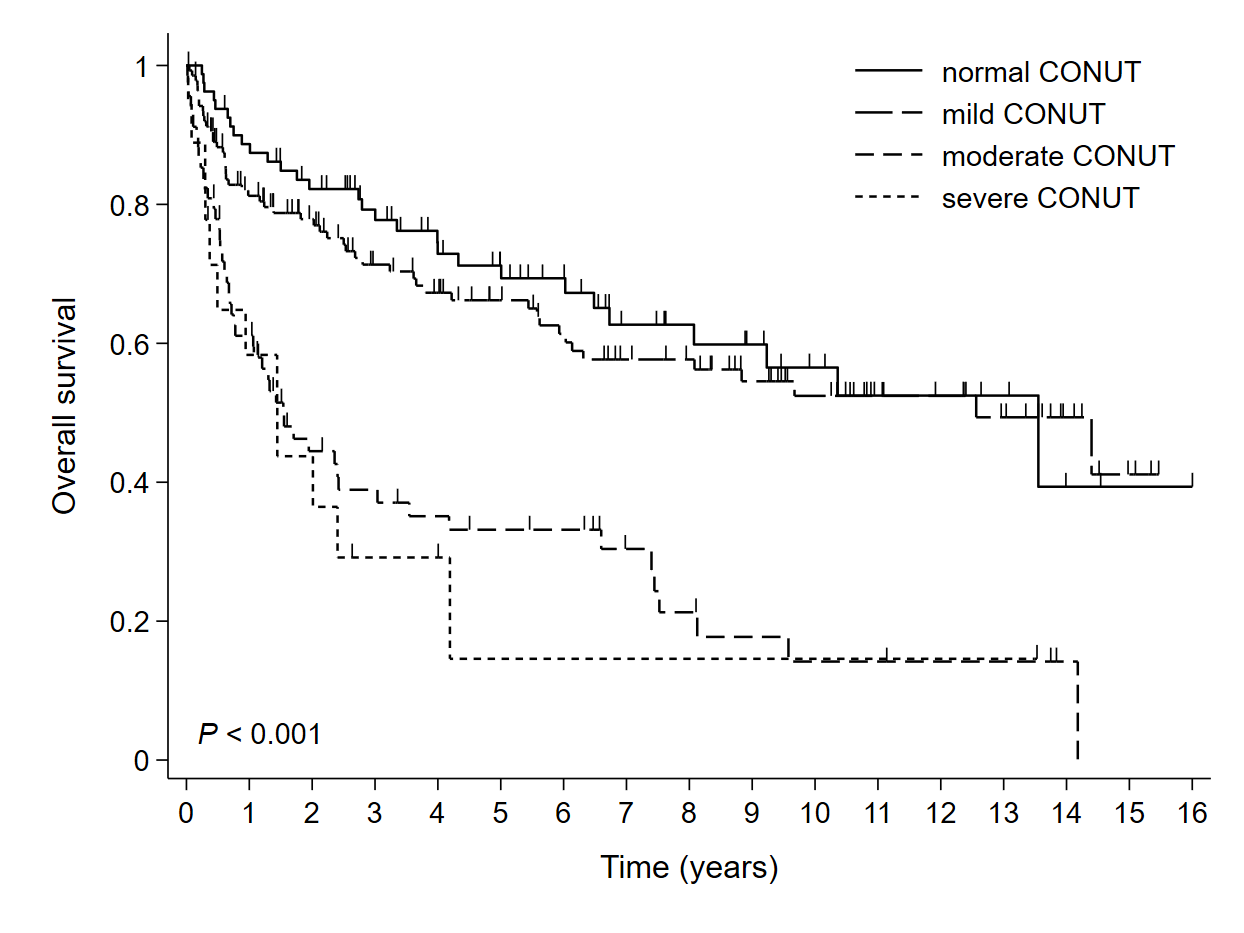


**Supplemental Fig. 2** Kaplan-Meier survival curves for overall survival according to the dose reduction from the first cycle and CONUT-SMI category in (A) group A, (B) group B, and (C) group C, respectively. *CONUT* Controlling Nutritional Status, *SMI* skeletal muscle index


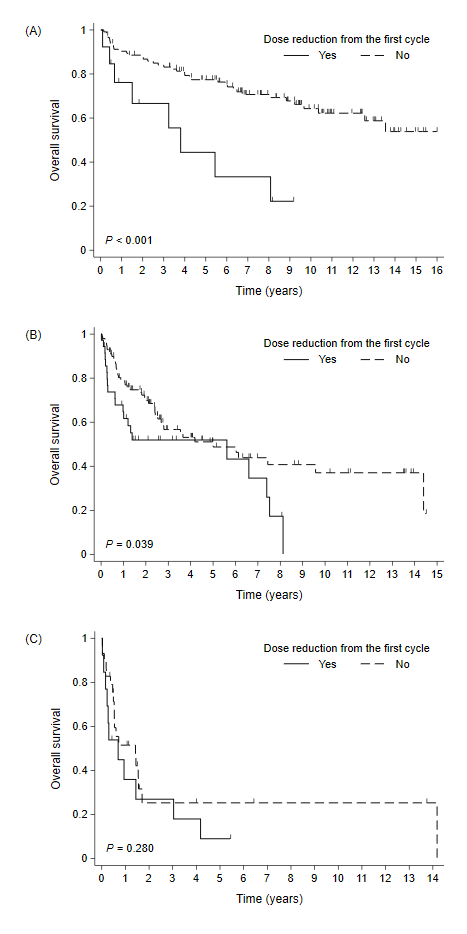


**Supplementary Table 1A** Bootstrap internal validation (1,000 replications) results for multivariate Cox regression analysis of progression-free and overall survival

| Progression-free survival | Observed HR | Bootstrap SE | Normal-based 95% CI | | *P* |
| --- | --- | --- | --- | --- | --- |
| Symptom stage (B vs. A) | 1.555 | 0.330 | 1.026 | 2.356 | 0.037 |
| NCCN-IPI |  |  |  |  |  |
| Low to low-intermediate | Ref. |  |  |  |  |
| High-intermediate | 4.754 | 1.223 | 2.871 | 7.871 | <0.001 |
| High | 8.190 | 2.522 | 4.479 | 14.974 | <0.001 |
| Bone marrow involvement (yes vs. no) | 1.109 | 0.274 | 0.683 | 1.801 | 0.676 |
| L3-SMI (low vs. high) | 1.561 | 0.283 | 1.094 | 2.228 | 0.014 |
| CONUT |  |  |  |  |  |
| Normal to mild | Ref. |  |  |  |  |
| Moderate to severe | 1.499 | 0.269 | 1.054 | 2.131 | 0.024 |
| Overall survival | Observed HR | Bootstrap SE | Normal-based 95% CI | | *P* |
| Symptom stage (B vs. A) | 1.556 | 0.332 | 1.024 | 2.364 | 0.038 |
| NCCN-IPI |  |  |  |  |  |
| Low to low-intermediate | Ref. |  |  |  |  |
| High-intermediate | 4.369 | 1.125 | 2.638 | 7.237 | <0.001 |
| High | 9.687 | 3.027 | 5.251 | 17.871 | <0.001 |
| Bone marrow involvement (yes vs. no) | 1.030 | 0.274 | 0.612 | 1.733 | 0.912 |
| L3-SMI (low vs. high) | 1.670 | 0.300 | 1.175 | 2.375 | 0.004 |
| CONUT |  |  |  |  |  |
| Normal to mild | Ref. |  |  |  |  |
| Moderate to severe | 1.470 | 0.287 | 1.003 | 2.154 | 0.048 |

*HR* hazard ratio, *SE* standard error, *95% CI* 95% confidence interval, *NCCN-IPI* National Comprehensive Cancer Network International Prognostic Index, *SMI* skeletal muscle index, *CONUT* Controlling Nutritional Status

**Supplementary Table 1B** Bootstrap internal validation (1,000 replications) results for multivariate Cox regression analysis of progression-free and overall survival

| Progression-free survival | Observed HR | Bootstrap SE | Normal-based 95% CI | | *P* |
| --- | --- | --- | --- | --- | --- |
| Symptom stage (B vs. A) | 1.556 | 0.322 | 1.038 | 2.334 | 0.032 |
| NCCN-IPI |  |  |  |  |  |
| Low to low-intermediate | Ref. |  |  |  |  |
| High-intermediate | 4.707 | 1.194 | 2.863 | 7.739 | <0.001 |
| High | 8.168 | 2.455 | 4.532 | 14.721 | <0.001 |
| Bone marrow involvement (yes vs. no) | 1.111 | 0.275 | 0.683 | 1.806 | 0.671 |
| CONUT + L3-SMI model |  |  |  |  |  |
| Group A | Ref. |  |  |  |  |
| Group B | 1.632 | 0.312 | 1.121 | 2.375 | 0.011 |
| Group C | 2.314 | 0.634 | 1.352 | 3.960 | 0.002 |
| Overall survival | Observed HR | Bootstrap SE | Normal-based 95% CI | | *P* |
| Symptom stage (B vs. A) | 1.544 | 0.323 | 1.025 | 2.326 | 0.038 |
| NCCN-IPI |  |  |  |  |  |
| Low to low-intermediate | Ref. |  |  |  |  |
| High-intermediate | 4.266 | 1.094 | 2.580 | 7.054 | <0.001 |
| High | 9.486 | 2.778 | 5.343 | 16.841 | <0.001 |
| Bone marrow involvement (yes vs. no) | 1.037 | 0.256 | 0.639 | 1.683 | 0.883 |
| CONUT-SMI category |  |  |  |  |  |
| Group A | Ref. |  |  |  |  |
| Group B | 1.721 | 0.339 | 1.170 | 2.533 | 0.006 |
| Group C | 2.423 | 0.669 | 1.410 | 4.162 | 0.001 |

*HR* hazard ratio, *SE* standard error, *95% CI* 95% confidence interval, *NCCN-IPI* National Comprehensive Cancer Network International Prognostic Index, *CONUT* Controlling Nutritional Status, *SMI* skeletal muscle index
